# Methicillin-Resistant Staphylococcus aureus Cellulitis Causing Meningitis From Hematogenous Dissemination: A Case Report

**DOI:** 10.7759/cureus.52969

**Published:** 2024-01-25

**Authors:** Omar M Masarweh, Suhail Saad-Omer, Michael Rohr, Neha Meda, Nicole Brenner

**Affiliations:** 1 Internal Medicine, University of Central Florida, Kissimmee, USA; 2 Internal Medicine, University of Central Florida College of Medicine, Orlando, USA

**Keywords:** infectious diseases, meningitis, cellulitis, septic emboli, valvular endocarditis, methicillin resistant staphylococcus aureus (mrsa)

## Abstract

Methicillin-resistant *Staphylococcus aureus* meningitis is commonly associated with surgical procedures that closely interact with the central nervous system; however, hematogenous spread via bacteremia is rarely reported. Here, we present a case of methicillin-resistant *Staphylococcus aureus* meningitis as a complication of a diabetic foot infection that disseminated into a bloodstream infection causing infective endocarditis, discitis, vertebral osteomyelitis, and meningitis that was successfully treated with intravenous daptomycin and rifampin.

## Introduction

Bacterial meningitis is most commonly caused by *Streptococcus pneumoniae, Neisseria meningitides,* and* Listeria monocytogenes.* Since the advent of antibiotics, treatment success rates have led to a favorable prognosis; however, it can still be life-threatening. Methicillin-resistant *Staphylococcus aureus* (MRSA) is a less frequently implicated bacteria, accounting for 1-9% of cases, and is associated with a high mortality rate [1]. Often associated with neurosurgical interventions, MRSA meningitis is rarely seen from hematogenous spread without some disruption to the central nervous system barrier. Traditionally, MRSA infections have been treated with agents such as vancomycin; however, due to its poor central nervous system (CNS) penetration, different agents such as linezolid and daptomycin have been reportedly used with good efficacy and total clearance of infection [1]. Herein, we present a case of MRSA meningitis, in which skin and soft-tissue infection was the nidus for infection leading to dissemination via hematogenous spread to the CNS.

## Case presentation

A 70-year-old man with a history of hypertension, hyperlipidemia, type 2 diabetes mellitus, subdural hematoma with evacuation 10 years prior, chronic osteomyelitis of the left foot resulting in amputation two years prior, and native-valve infective endocarditis (IE) status post bioprosthetic valve replacement six years prior presented to the emergency department for four days of fever, headaches, and lethargy. Headaches were described as tension-like but more intense than his typical headaches without associated nausea, vomiting, photophobia, or neck stiffness.

On initial assessment, his temperature was 100.4°F, blood pressure was 131/82 mmHg, heart rate was 115 beats per minute, and oxygen saturation was 93% on room air. On physical examination, he was lethargic and slow to respond to verbal and physical stimuli. He was oriented only to name. His neck was supple and no meningismus was noted. Non-tender pitting edema and warmth in the left lower extremity without open wounds or active drainage were noted. Laboratory investigation revealed normocytic anemia with hemoglobin of 9.7 mg/dL, thrombocytopenia with a platelet (PLT) count of 97,000/mm^3^, and a white blood cell (WBC) count of 4,400/mm^3^, with 87% neutrophils (reference 34-68%) and 4.8% immature granulocytes (reference 0.0-0.4%). A complete metabolic panel showed acute kidney injury with a serum creatinine of 2.15 mg/dL and a baseline from two years prior of 0.84 mg/dL. The lactic acid level was elevated at 3.5 mmol/L. Coagulation evaluation showed a prolonged prothrombin time (PT) of 15.8 seconds. Urinalysis was significant for proteinuria (100 mg/dL), microscopic hematuria (11-25 RBC/hpf), and pyuria (11-25 WBC/hpf). Work-up for deep venous thrombosis revealed substantially elevated D-dimer levels (56,025 ng/mL) with computed tomography (CT) with angiography of the chest negative for pulmonary embolism and bilateral lower-extremity ultrasounds negative for deep vein thrombosis. An electrocardiogram was significant for an accelerated junctional rhythm. CTs of the head, abdomen, and pelvis were negative for any acute pathologies, and CT of the left ankle and foot demonstrated soft-tissue swelling without signs of osteomyelitis. The patient was started on intravenous fluids, vancomycin, and piperacillin/tazobactam.

Hours later, the patient's mental status deteriorated, and was subsequently intubated for airway protection due to a Glasgow coma score of 5. Arterial blood gas showed respiratory alkalosis with compensatory metabolic acidosis (pH = 7.48, pCO_2_ = 23 mmHg, and bicarbonate = 17 mmol/L). Repeat labs revealed worsening anemia (hemoglobin = 7.7 mg/dL) and thrombocytopenia (PLT = 77,000/mm^3^). WBC count remained unchanged. Inflammatory markers were elevated (erythrocyte sedimentation rate = 81 mm/h, C-reactive protein = 31 mg/dL) and the coagulation panel showed prolonged PT (15.6 s), partial thromboplastin time (PTT) (39 s), and high serum fibrinogen (733 mg/dL). Further work-up for disseminated intravascular coagulation and thrombotic thrombocytopenic purpura was negative for hemolytic anemia; reticulocyte count was normal (0.7%), haptoglobin (416.2 mg/dL) and lactate dehydrogenase (284 U/L) were elevated, and ADAMTS13 activity (33.8%) was only slightly reduced. Bilirubin levels were normal and no schistocytes were seen on peripheral blood smear. It was determined that the hematologic findings were consistent with sepsis as blood and left lower-extremity wound cultures resulted positive for MRSA the following day. 

**Figure 1:**
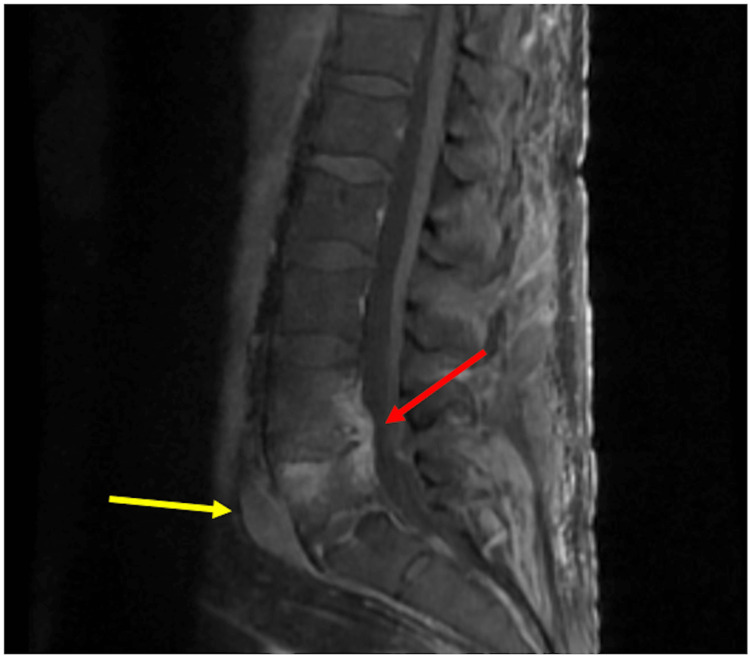
Magnetic resonance imaging of the lumbar spine showing partial destruction of the L4-L5 intervertebral disc with enhancement of the mucous pulposis (red arrow) and L5 phlegmon (yellow arrow).

**Figure 2:**
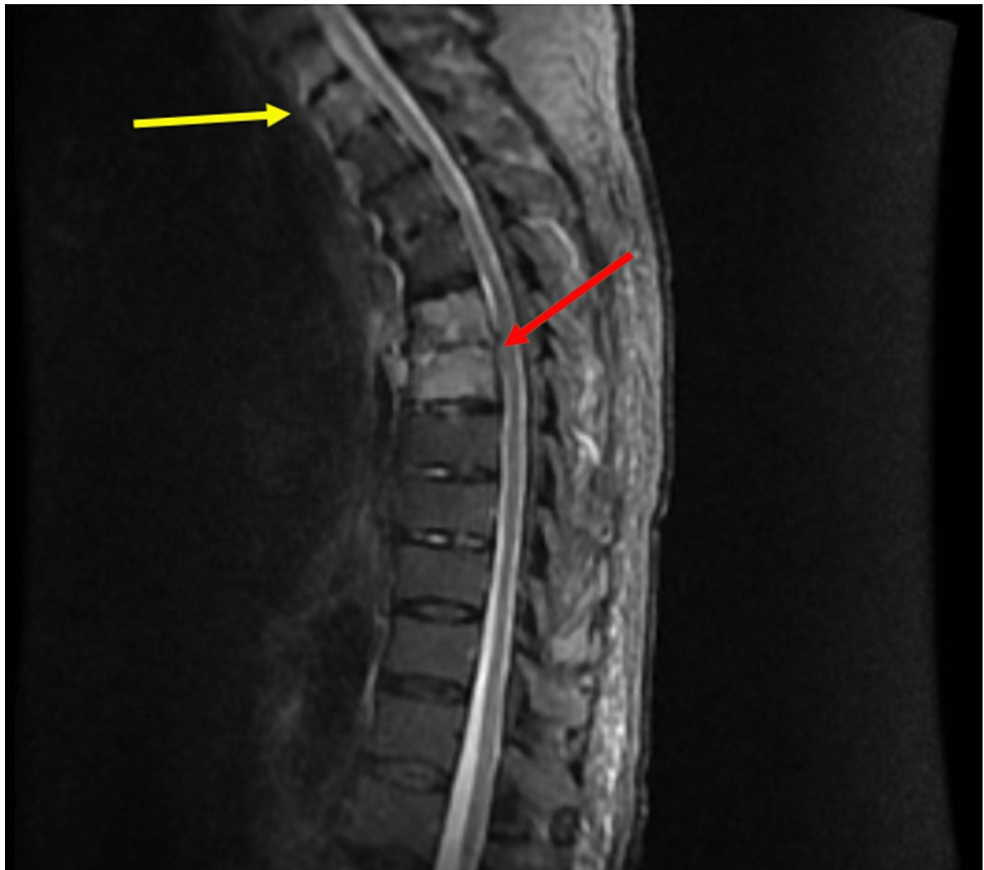
Magnetic resonance imaging of thoracic spine showing osteomyelitis/discitis at T6-T7 (red arrow) and likely osteomyelitis at T1 and T2 (yellow arrow).

Due to the worsening mental status, a meningitis work-up was pursued. Lumbar puncture demonstrated hazy cerebrospinal fluid (CSF), CSF WBC = 537/µL, CSF neutrophils = 92.9%, CSF red blood cells = 49/mm^3^, CSF lymphocytes = 7.1%, CSF glucose = 161 mg/dL, CSF protein = 101 mg/dL and gram-positive cocci in clusters on CSF gram stain (Table 1), which later speciated to MRSA. The initial transthoracic echocardiogram failed to demonstrate valvular vegetations; however, a subsequent transesophageal echocardiogram showed extensive mitral valve vegetations. Magnetic resonance imaging (MRI) of the brain showed a few areas of acute infarcts in bilateral frontoparietal lobes consistent with septic emboli. MRI of the cervical, thoracic, and lumbar spine also showed discitis with osteomyelitis at levels T6 and T7 (Figure 1) and L4-L5 (Figure 2). Multidisciplinary team discussion with cardiothoracic surgery, cardiology, infectious disease, and the patient’s medical decision maker concluded that no surgical interventions would be undertaken, and medical management would be attempted first, with repeat evaluation if clinical circumstances changed.

**Table 1:** Cerebral spinal fluid analysis. VDRL, Venereal Disease Research Laboratory.

Spinal Fluid	Value	Reference Range
Appearance	Hazy	
Glucose	161 mg/dL	40-70 mg/dL
Protein	101 mg/dL	15-45 mg/dL
Total cell count	100 cells	0-100 cells
White blood cells	5,515	
Neutrophils	90.5%	
Lymphocytes	9.5%	
Red blood cells	47/mm^3^	
VDRL	Negative	
Cryptococcus antigen	Negative	
Gram stain	Gram-positive cocci in clusters	

It is believed that the nidus of the patient’s MRSA meningitis was left leg cellulitis that disseminated into a bloodstream infection causing IE, osteomyelitis, discitis, and meningitis. The final treatment regimen consisted of intravenous daptomycin and oral rifampin for four weeks; vancomycin was discontinued after three weeks due to worsening renal function. The patient markedly improved after the diagnosis was made and optimal therapy was initiated. He was discharged home with a follow-up planned. 

## Discussion

Community-acquired bacterial meningitis in developed countries is most commonly due to *Streptococcus pneumoniae, Neisseria meningitides*, and *Listeria monocytogenes,* which primarily occurs in patients over 50 years of age or individuals with impaired cell-mediated immunity [2]. Staphylococcal meningitis, while rare, is a potentially life-threatening condition. Previous studies have shown that in the United States, *Staphylococcus aureus* (*S. aureus*) meningitis accounts for 1-3% of cases of meningitis, whereas worldwide, *S. aureus* meningitis constitutes 0.3-8.8% of all cases [3]. Despite this seemingly low prevalence, recent studies have revealed that there seems to be an increasing rate of MRSA meningitis in the United States. One study conducted in Detroit, Michigan, revealed that among 668 cases of bacterial meningitis, 33 (4.9%) were found to be positive for MRSA meningitis [4].

MRSA meningitis is usually thought of as comprising two separate entities based on the etiology of the infection. It is usually divided into postoperative MRSA meningitis and spontaneous MRSA meningitis. In postoperative MRSA meningitis, bacteria are typically introduced following neurosurgical procedures, CSF device placement, or through trauma. Spontaneous MRSA meningitis, however, typically occurs after bacteria is disseminated systemically secondary to staphylococcal infection outside the CNS [5]. Postoperative MRSA meningitis forms the vast majority of cases with one case series in Spain showing that among 86 MRSA meningitis patients, 78 (91%) of them were secondary to postoperative MRSA meningitis. Among the 78 patients with postoperative meningitis, the most common predisposing conditions were CSF devices (74%), neurosurgery (45%), CSF leakage (17%), and head trauma (12%). These patients are typically younger and without chronic comorbidities [6]. On the other hand, spontaneous MRSA meningitis typically occurs in patients with severe underlying conditions such as diabetes, chronic kidney disease, cardiovascular disease, and HIV. These patients frequently visit the hospital due to complications of these conditions and spontaneous MRSA meningitis could therefore be thought of as a healthcare-associated infection. Healthcare-associated meningitis is defined as an infection that was not present on admission but that was contracted no sooner than 48 hours after admission or up to one month after hospital discharge. Patients with spontaneous meningitis are typically older with one case series of 34 MRSA meningitis patients showing that patients with spontaneous meningitis had a significantly older average age (55.8 years vs 31.93; p = 0.021) [6]. Possible etiologies for hematogenous CNS infection for those with MRSA meningitis include those due to dissemination from distant sources such as epidural abscesses, endocarditis, skin and soft-tissue infection, and pneumonia. Studies have shown that bacteremia and extra-CNS infections were seen more frequently in hematogenous than in postoperative meningitis [5]. In a Danish nationwide study, a significant number of patients with *S. aureus* meningitis (60%) had secondary foci such as endocarditis (36%) or osteomyelitis (16%) [7]. This is consistent with our case, where chronic osteomyelitis and suppurative arthritis, undetected at first, were likely secondary to the patient’s overall diminished immune system. Therefore, the crucial moment in diagnosing and subsequently treating our patient was the accurate identification of the inflammatory foci.

Although the clinical presentations of postoperative and spontaneous meningitis are similar, focal neurologic deficit and septic shock are present significantly more frequently in patients with spontaneous infections. The higher frequency of septic shock in spontaneous meningitis has been reported in a case series of *S. aureus* meningitis [8]. Despite its increasing occurrence, there still remains no general consensus for the treatment of MRSA meningitis. The Infectious Diseases Society of America has recommended two weeks of vancomycin therapy for MRSA meningitis. The efficacy of vancomycin therapy, however, is limited due to its poor penetration into CSF, with an estimated penetration of only 1% and 5% with uninflamed and inflamed meninges, respectively [9]. To circumvent poor CNS antibiotic penetration, various studies have suggested the use of rifampicin for synergistic effect, which was used in our patient [10]. Other studies have also explored the possibility of using intravenous (IV) linezolid. One case series compared the use of IV linezolid and IV vancomycin and found that IV linezolid was superior to IV vancomycin in patients with a high vancomycin minimum inhibitory concentration [11]. Another study reviewed the use of IV teicoplanin, suggesting this agent may be an alternative to vancomycin in treating these cases [12]. Our patient responded well to vancomycin; however, due to worsening renal function, his antibiotic regimen was transitioned to daptomycin which was well tolerated with eventual resolution and complete clearance of the infection.

## Conclusions

MRSA meningitis is a rare entity that is usually brought on by disruption to the CNS via neurosurgical intervention. However, multiple cases of MRSA meningitis from hematogenous spread from primary infections outside the CNS have been reported. MRSA meningitis requires prompt identification and rapid initiation of treatment as mortality rates remain high. Further research is needed to assess the bacteria’s ability to penetrate the CNS and other effective antibiotic regimens as suitable alternatives to vancomycin due to its relatively low CNS penetration and risk for nephrotoxicity.
